# MRI-Based Morphologic Patterns of the Peroneus Quartus in a Korean Clinical Cohort: An Anatomical Study with an Illustrative Surgical Case

**DOI:** 10.3390/jcm15197732

**Published:** 2026-10-08

**Authors:** Otgonsaikhan Nomkhondorj, Ye-Chan Choi, Jeong-Hyun Park, Hyung-Wook Kwon, Digud Kim, Yu-Jin Choi, Yunil Choe, Kwang-Rak Park, Jaeho Cho

**Affiliations:** 1 Department of Orthopaedic Surgery, Chuncheon Sacred Heart Hospital, Hallym University College of Medicine, Chuncheon 24253, Republic of Korea; 2 Department of Anatomy & Cell Biology, College of Medicine, Kangwon National University, Chuncheon 24341, Republic of Korea; jhpark@kangwon.ac.kr (J.-H.P.);; 3 Department of Anatomy, College of Medicine, Dongguk University, Gyeongju 38066, Republic of Korea; 4 Institute of New Frontier Research Team, Hallym University, Chuncheon 24253, Republic of Korea

**Keywords:** peroneus quartus, fibularis quartus, magnetic resonance imaging, anatomical variation, peroneal tendon, lateral ankle pain, clinical anatomy, surgical anatomy

## Abstract

**Background/Objectives:** The peroneus quartus (PQ) is an accessory musculotendinous structure of the lateral leg with variable origin and distal attachment. Although its anatomy has been described in cadaveric and imaging studies, the morphologic patterns encountered on routine ankle MRI remain clinically relevant. This study aimed to characterize MRI-visible PQ morphology in a Korean clinical cohort and to compare the observed patterns with previously reported anatomical variants. An illustrative surgically treated case was also reviewed. **Methods:** Three hundred ankle MRI examinations obtained between February 2020 and February 2024 were reviewed. After exclusion of 33 examinations in which the peroneal compartment could not be reliably assessed, 267 unique patients were included. Two readers independently evaluated PQ presence and distal attachment using predefined MRI criteria. Interobserver agreement was assessed with Cohen’s kappa. The surgically treated case was reviewed separately as an illustrative clinical correlation. **Results:** A PQ was identified in 41 of 267 ankles (15.4%; 95% CI, 11.5–20.2%). The proximal origin was directly visualized in 32 PQ-positive examinations, arising from the peroneus brevis in 19 and the distal fibula in 13; in nine examinations, the origin was outside the MRI field of view. Thirty-five PQs (85.4%) inserted into the retrotrochlear calcaneal region, whereas six (14.6%) joined the peroneus longus tendon distally. Interobserver agreement was complete for both PQ detection and distal attachment classification (κ = 1.00). In the illustrative case, a symptomatic ganglion was associated with an operatively confirmed degenerative PQ tendon tear. **Conclusions:** The PQ was identified in 15.4% of ankle MRIs in this selected Korean clinical cohort and showed two predominant MRI-visible distal attachment patterns. Most inserted into the retrotrochlear calcaneal region, whereas a smaller proportion joined the peroneus longus tendon. These findings describe MRI-visible anatomy rather than the full anatomical spectrum. The surgical case further illustrates that symptomatic pathology may involve the PQ tendon and its adjacent sheath.

## 1. Introduction

The peroneus quartus (PQ), also termed the fibularis quartus, is an accessory musculotendinous structure of the lateral compartment of the leg. It has attracted longstanding anatomical interest because of its highly variable origin, insertion, and morphology and the resulting complexity of its nomenclature [1,2,3,4]. The PQ most commonly arises from the peroneus brevis (PB), although origins from the distal fibula, peroneus longus (PL), intermuscular septum, and combinations of these structures have also been described [2,3,4,5]. Its distal attachment is even more variable and may involve the retrotrochlear eminence or peroneal trochlea of the calcaneus, cuboid, fifth metatarsal, PB or PL tendon, or adjacent retinacular structures [3,4,5,6,7,8,9].

Historically, the terminology applied to accessory peroneal muscles has been based primarily on their distal insertion. Terms such as peroneocalcaneus externum, peroneocuboideus, and peroneoperoneolongus have therefore been used to describe configurations [4,6,8]. This anatomical diversity has made standardization difficult. A systematic review and meta-analysis by Yammine reported an MRI prevalence of 10.6%, with the PB representing the most frequent origin and the retrotrochlear region of the calcaneus the most frequent distal attachment [10]. Importantly, a cadaveric study of Korean adults by Hur et al. identified fibularis quartus muscles in 16.3% of specimens and demonstrated marked variation in both the form of origin and site of insertion [8].

Magnetic resonance imaging provides an opportunity to evaluate PQ morphology in living individuals while preserving the intact spatial relationships of the accessory musculotendinous unit to the PB, PL, distal fibula, retromalleolar groove, and calcaneus [3,11]. Nevertheless, routine ankle MRI has intrinsic limitations: the proximal origin may fall outside the imaging field of view, and thin distal tendinous attachments may be difficult to distinguish from adjacent structures [11]. Consequently, the morphology visualized on clinical MRI may not necessarily reproduce the full spectrum observed during anatomical dissection.

The clinical importance of the PQ has also evolved from that of an anatomical curiosity to a potentially relevant component of peroneal tendon disorders. The additional musculotendinous structure may increase occupancy of the retromalleolar fibro-osseous space and has been implicated in chronic lateral ankle pain, tendon subluxation, tenosynovitis, and longitudinal PB tendon tears [3,7,12]. Although earlier studies yielded conflicting evidence regarding this association [10], more recent MRI investigations have suggested a relationship between PQ and peroneal tendon pathology. Pota et al. identified the PQ in 13.8% of 849 ankle MRIs [13]. Yuksel et al. evaluated 1160 ankle MRIs and reported a PQ prevalence of 10.6%, with significantly increased odds of PB tendinitis and PB tear in PQ-positive ankles [14]. A 2026 imaging-based systematic review and meta-analysis subsequently estimated a pooled PQ prevalence of approximately 11% and reported significantly greater odds of peroneal tendon tears in ankles with a PQ [15].

Most PQ muscles are asymptomatic, and their significance depends on the clinical context and associated imaging abnormalities [11,15]. A recent review of supernumerary muscles of the leg and foot further emphasizes that these variants may be clinically relevant when they mimic or contribute to symptomatic conditions and should be recognized on imaging [16]. Published surgical reports have emphasized not only symptomatic crowding but also the potential use of the PQ tendon as local autologous tissue for reconstruction of the superior peroneal retinaculum or augmentation of peroneal tendon repair [7,17].

Therefore, the aim of this study was to characterize the MRI-visible morphologic patterns of the peroneus quartus in a selected Korean clinical cohort and to compare these imaging findings cautiously with previously reported anatomical patterns. In addition, an illustrative surgically treated case was reviewed to demonstrate the potential clinical relevance of a symptomatic lesion in the PQ region without implying systematic operative validation of the imaging cohort.

## 2. Materials and Methods

This retrospective imaging-based anatomical study was performed at Chuncheon Sacred Heart Hospital, Hallym University College of Medicine. The study was conducted in accordance with the Declaration of Helsinki and was approved by the Institutional Review Board of Chuncheon Sacred Heart Hospital (IRB No. CHUNCHEON 2024-06-007; approval date, 28 June 2024). All MRI examinations included in the retrospective cohort had been acquired before IRB approval because the corrected study inclusion period was February 2020 through February 2024. Individual research consent was waived for the retrospective MRI cohort. Written informed consent for publication of the clinical information and images was obtained separately from the patient included in the illustrative surgical case.

### 2.1. Patient Selection

Ankle MRI examinations obtained between February 2020 and February 2024 were identified from the institutional PACS. The source population consisted of patients who underwent ankle MRI for clinical evaluation of suspected Achilles tendon injury. All 300 patients in whom no Achilles tendon injury was identified on MRI constituted the initial study cohort; therefore, the number 300 did not represent an arbitrary numerical limit or a randomly selected subset. Eligibility was assessed by O.N. and J.C. before the formal PQ morphology reading. Examinations were excluded only when (1) trauma, prior surgery, tumor, infection, marked edema, or another lesion involving the lateral ankle or distal lower leg substantially distorted the peroneal compartment and prevented reliable PQ assessment, or (2) inadequate image quality or insufficient anatomic coverage prevented reliable evaluation of the PQ. Among the 300 initially eligible examinations, 33 were excluded: 13 because of anatomical distortion and 20 because of inadequate image quality or insufficient anatomic coverage. Consequently, 267 examinations from 267 unique patients, with one ankle examination per patient, were included in the final analysis. No patient contributed to bilateral examinations or repeated MRI examinations. The final cohort had a mean age of 43.6 years and included 242 male patients (90.6%) and 25 female patients (9.4%); 162 examinations were of the right ankle and 105 of the left ankle. Because this was a selected clinical referral cohort rather than a population-based sample, the marked male predominance and referral setting should be considered when interpreting prevalence and morphology.

### 2.2. MRI Acquisition and Evaluation of the Peroneus Quartus

MRI examinations were performed using one of two 3.0-T MRI systems: a Philips Ingenia (Philips Healthcare) with a dedicated 8-channel SENSE ankle coil or a MAGNETOM Skyra (Siemens Healthineers) with a dedicated 16-channel foot-and-ankle coil. All study examinations were non-contrast routine ankle MRIs obtained in axial, sagittal, and coronal planes. The Philips protocol included T2-weighted Dixon acquisitions with water and in-phase reconstructions and T1-weighted turbo spin-echo (TSE) sequences. The sequence-specific field of view (FOV) and acquisition matrix were as follows: sagittal T2 Dixon, 180 × 180 mm and 376 × 333; sagittal T1 TSE, 180 × 180 mm and 464 × 458; axial T2 Dixon, 140 × 140 mm and 380 × 320; axial T1 TSE, 140 × 140 mm and 480 × 324; coronal T2 Dixon, 180 × 180 mm and 488 × 448; and coronal T1 TSE, 180 × 180 mm and 476 × 464. The Siemens protocol included T2-weighted TSE sequences with and without fat suppression and T1-weighted TSE sequences. The corresponding FOVs and matrices were: sagittal T2 TSE FS, 200 × 200 mm and 448 × 314; sagittal T2 TSE, 200 × 200 mm and 384 × 269; sagittal T1 TSE, 200 × 200 mm and 448 × 269; axial T2 TSE FS, 150 × 150 mm and 384 × 269; axial T2 TSE, 150 × 150 mm and 448 × 269; axial T1 TSE, 150 × 150 mm and 384 × 250; coronal T2 TSE FS, 210 × 210 mm and 320 × 320; coronal T2 TSE, 210 × 210 mm and 448 × 269; and coronal T1 TSE, 210 × 210 mm and 384 × 230. All sequences were acquired with a 3.0 mm slice thickness and 0.3 mm interslice gap. Anatomical coverage extended approximately from the distal one-fourth of the lower leg to the proximal one-third of the metatarsals. MRI examinations were evaluated using predefined criteria. A PQ was defined as a distinct accessory musculotendinous structure that could be separately delineated from the peroneus brevis (PB) and peroneus longus (PL) and followed on sequential images, with orthogonal-plane confirmation whenever available. A low-lying PB muscle belly was distinguished by continuity with the main PB musculotendinous unit without formation of a separate accessory course, and a longitudinal split of the PB or PL tendon was not classified as a PQ. Proximal origin and distal attachment were assessed separately and considered confirmed only when anatomical continuity could be directly followed. If the proximal extent was outside the field of view, origin was recorded as not directly visualized. Calcaneal attachment was defined as a distinct insertion on the lateral calcaneus in the retrotrochlear region posterior to the peroneal trochlea. PL attachment required direct continuity on sequential images; mere close contact was insufficient. Uncertain distal attachments were recorded as indeterminate, and multiple slips or attachments were recorded separately. MRI examinations were independently re-reviewed by the same two board-certified orthopedic surgeons who had assessed them previously (O.N. and J.C.), with 8 and 16 years of post-certification clinical experience, respectively. During this re-review, the readers were blinded to each other’s interpretations, the original MRI reports, and the patients’ clinical information. Interobserver agreement for PQ detection was assessed across all 267 examinations using unweighted Cohen’s kappa, observed percentage agreement, and a reader cross-tabulation. Agreement for distal attachment classification was assessed separately among the 41 examinations independently classified as PQ-positive by both readers. The agreement analyses used the initial classifications recorded during this re-review, before any joint discussion, regardless of the readers’ confidence levels. Examinations for which either reader reported lower confidence were flagged and reviewed jointly only after completion of the agreement analyses to establish the final morphologic classification.

### 2.3. Clinical Case Review

In addition to the imaging cohort, a surgically treated patient with a symptomatic lesion in the PQ region was reviewed separately as an illustrative clinical case; this patient was not included in the 267-patient imaging cohort because the adjacent cystic lesion distorted the local anatomy. Clinical records, MRI findings, operative findings, histopathologic results, and available postoperative follow-up were retrospectively evaluated. Attention was paid to the MRI and intraoperative appearance of the PQ tendon, its relationship to the cystic lesion and the PB and PL tendons, the documented tendon lesion, the operative procedure, and postoperative recurrence or complications. Written informed consent for publication of the clinical information and images was obtained from the patient.

### 2.4. Statistical Analysis

The present study was primarily descriptive. Continuous demographic data were summarized using mean ± standard deviation and range when available, whereas categorical findings were expressed as frequencies and percentages. PQ prevalence was calculated using the 267 unique patients/ankles as the denominator. Wilson 95% confidence intervals were reported for PQ prevalence and distal attachment patterns among evaluable PQ-positive examinations. Because no patient contributed bilateral or repeated examinations, each observation was treated as independent. Interobserver agreement was evaluated using observed percentage agreement, reader cross-tabulations, and unweighted Cohen’s kappa. Asymptotic 95% confidence intervals for kappa were not reported because complete observed agreement produced degenerate intervals of 1.00–1.00, which did not meaningfully quantify uncertainty. Data were analyzed using IBM SPSS Statistics version 23.0 for Windows (IBM Corp., Armonk, NY, USA).

## 3. Results

### 3.1. Study Population and Prevalence of the Peroneus Quartus

All 300 patients meeting the source-cohort criterion were initially considered. Thirty-three examinations were excluded because the peroneal compartment could not be reliably assessed: 13 because of anatomical distortion and 20 because of inadequate image quality or insufficient anatomic coverage. The final analysis included 267 MRI examinations from 267 unique patients and 267 unique ankles, with no bilateral or repeated examinations. The mean age was 43.6 ± 10.6 (20–75) years; 242 patients were male (90.6%) and 25 were female (9.4%), and 162 ankles were right-sided and 105 left-sided. A PQ was identified in 41 of 267 ankles, corresponding to a prevalence of 15.4% (95% CI, 11.5–20.2%) (Table 1).

Among the 41 PQ-positive patients, the mean age was 40.8 years; 31 were male and 10 were female, and 19 ankles were right-sided and 22 were left-sided. The readers agreed on PQ detection in all 267 examinations and on distal attachment classification in all 41 PQ-positive examinations (observed agreement, 100%; κ = 1.00 for both; Supplementary Table S1). Seven examinations were flagged as lower-confidence for distal attachment by one reader: three classified as PL attachment and four as calcaneal attachment. In each of these seven examinations, the other reader made the same classification with confidence. Joint review after the agreement analysis did not change any of these distal attachment classifications.

### 3.2. MRI-Based Morphologic Patterns

Among the 41 PQ-positive examinations, the proximal origin was directly visualized in 32 (78.0%): 19 arose from the peroneus brevis and 13 from the distal fibula. In the remaining nine examinations (22.0%), the proximal origin lay outside the MRI field of view and was recorded as not directly visualized. Among the 35 PQs with calcaneal attachment, 18 arose from the peroneus brevis, 12 from the distal fibula, and five had an origin that was not directly visualized. Among the six PQs with peroneus longus attachment, one arose from each structure, and four had an origin that was not directly visualized (Table 2). Given that the origin was directly visualized in only two examinations with peroneus longus attachment, these distributions were described without formal statistical comparison.

#### 3.2.1. Pattern 1: Calcaneal Insertion Pattern (Figure 1)

Distal attachment was classified in all 41 examinations based on direct visualization of the distal tendon course. Thirty-five demonstrated a calcaneal attachment pattern (85.4%; 95% CI, 71.6–93.1%), in which the distal PQ tendon could be followed to the retrotrochlear region of the lateral calcaneus. No distal attachment was classified as indeterminate, and no multiple distal slips or attachments were identified in the evaluable cohort.

**Figure 1:**
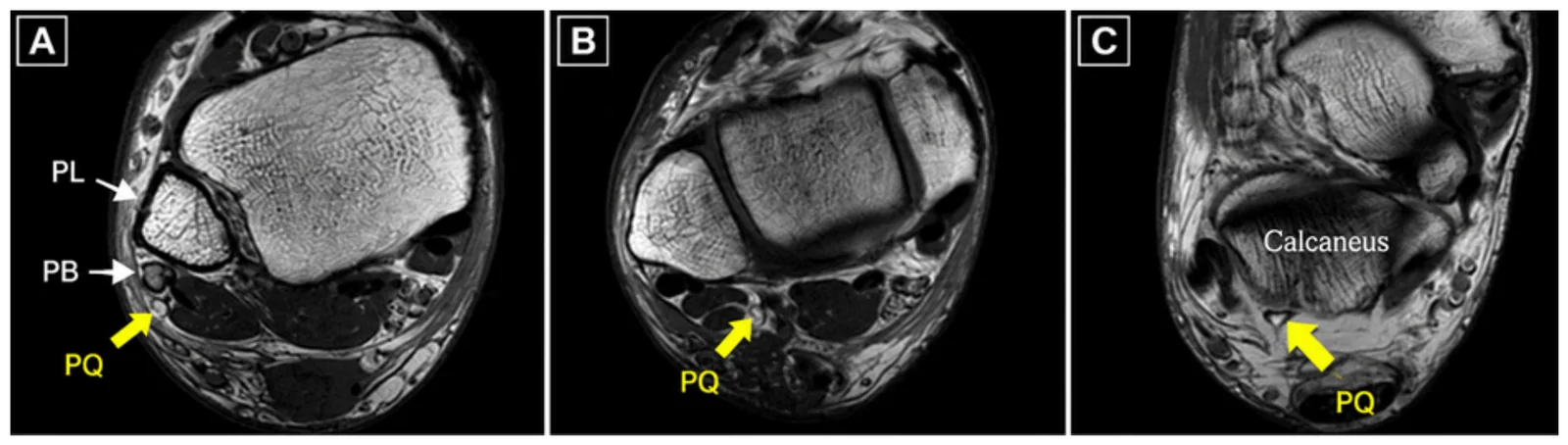
Representative MRI of the calcaneal insertion pattern of the peroneus quartus (PQ). Serial axial T1-weighted MR images (**A**–**C**), arranged from proximal to distal, demonstrate the PQ as a distinct tendon separate from the peroneus brevis (PB) and peroneus longus (PL) and its distal course toward the lateral calcaneus. (**A**) At a proximal level, the PQ (yellow arrow) is seen as a separate structure from the PB (white arrowhead) and PL (white arrowhead). (**B**) At an intermediate level, the PQ (yellow arrow) courses distally. (**C**) At a distal level, the PQ (yellow arrow) inserts onto the lateral calcaneus in the retrotrochlear region posterior to the peroneal trochlea.

#### 3.2.2. Pattern 2: Peroneus Longus Attachment Pattern (Figure 2) 

In 6 of 41 PQ-positive examinations (14.6%; 95% CI, 6.9–28.4%), the PQ tendon demonstrated direct distal continuity with the PL tendon on sequential images. Mere close apposition without demonstrable continuity was not sufficient for this classification. Because this pattern was represented by only six ankles, its observed frequency should be interpreted with appropriate uncertainty. On re-review of the 41 PQ-positive examinations, no consistent pattern of associated peroneal tendon abnormality or adjacent cystic lesion was identified. Because the study was not designed as a comparison with PQ-negative ankles, no statistical association between PQ and peroneal tendon pathology was assessed.

**Figure 2:**
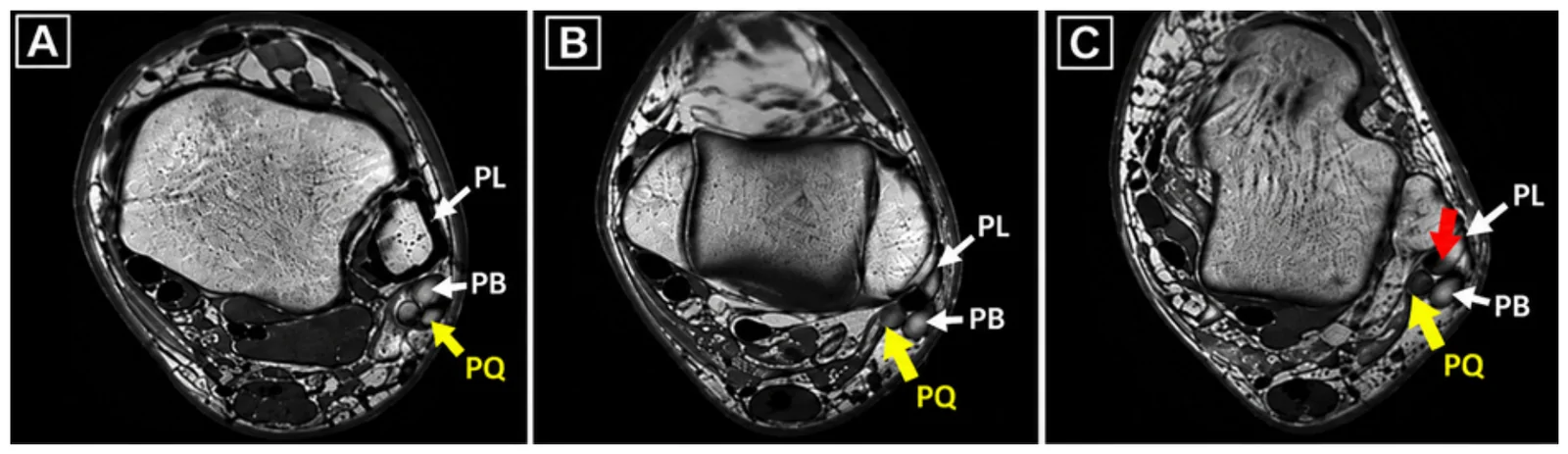
Representative MRI demonstrating distal confluence of the peroneus quartus (PQ) with the peroneus longus (PL) tendon. Serial axial T1-weighted MR images (**A**–**C**), arranged from proximal to distal, demonstrate the PQ as a distinct accessory tendon and its distal course toward the PL tendon. (**A**) The PQ is identified as a separate structure from the peroneus brevis (PB) and PL. (**B**) The PQ courses distally toward the PL tendon while remaining separately identifiable. (**C**) The PQ joins the PL tendon at the PQ–PL confluence (red arrow), demonstrating direct tendon continuity. The PQ, PB, and PL are labeled in each panel.

### 3.3. Clinical and Surgical Correlation

**Figure 3:**
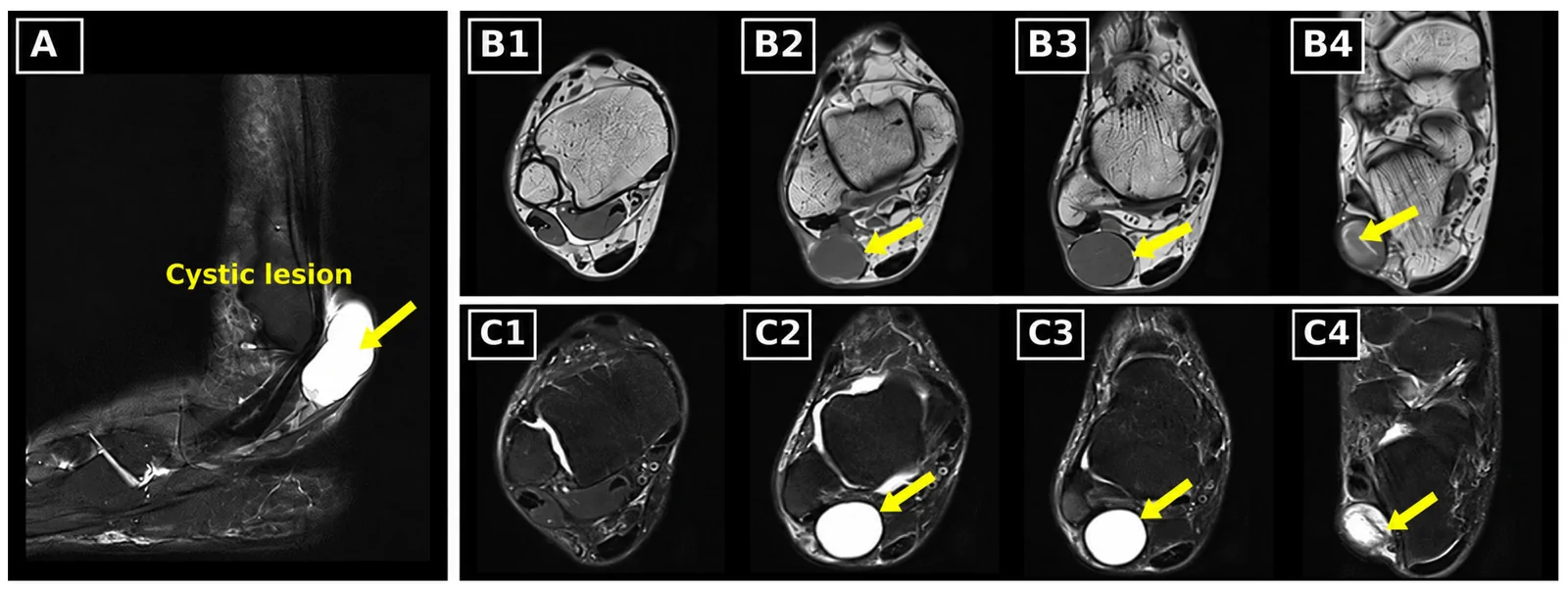
Preoperative MRI findings of the illustrative surgical case. (**A**) Sagittal fluid-sensitive fat-suppressed MR image demonstrates an approximately **3 × 2 cm^2^** well-defined cystic lesion in the posterolateral ankle adjacent to the peroneus quartus (PQ) tendon. (**B1**–**B4**) Serial images demonstrate the PQ tendon and its anatomical relationship to the cystic lesion. (**C1**–**C4**) Serial fat-suppressed images demonstrate the fluid-containing nature and extent of the lesion adjacent to the PQ. Based on the preoperative imaging findings, the differential diagnosis included a ganglion cyst and hematoma. Histopathological examination of the subsequently excised lesion confirmed a ganglion cyst. The arrows indicate the cystic lesion. Focal thinning and indistinctness of the adjacent PQ tendon were described on review of the preoperative MRI but are not specifically delineated by the arrows in these panels.

A 71-year-old man with a history of recurrent ankle sprains presented with progressive posterolateral ankle swelling and pain. MRI demonstrated an approximately 3 × 2 cm cystic lesion adjacent to the PQ tendon. The PQ tendon showed focal thinning and increased indistinctness/haziness on MRI, raising suspicion for degenerative tendon injury. The PB and PL tendons appeared intact on MRI (Figure 3).

**Figure 4:**
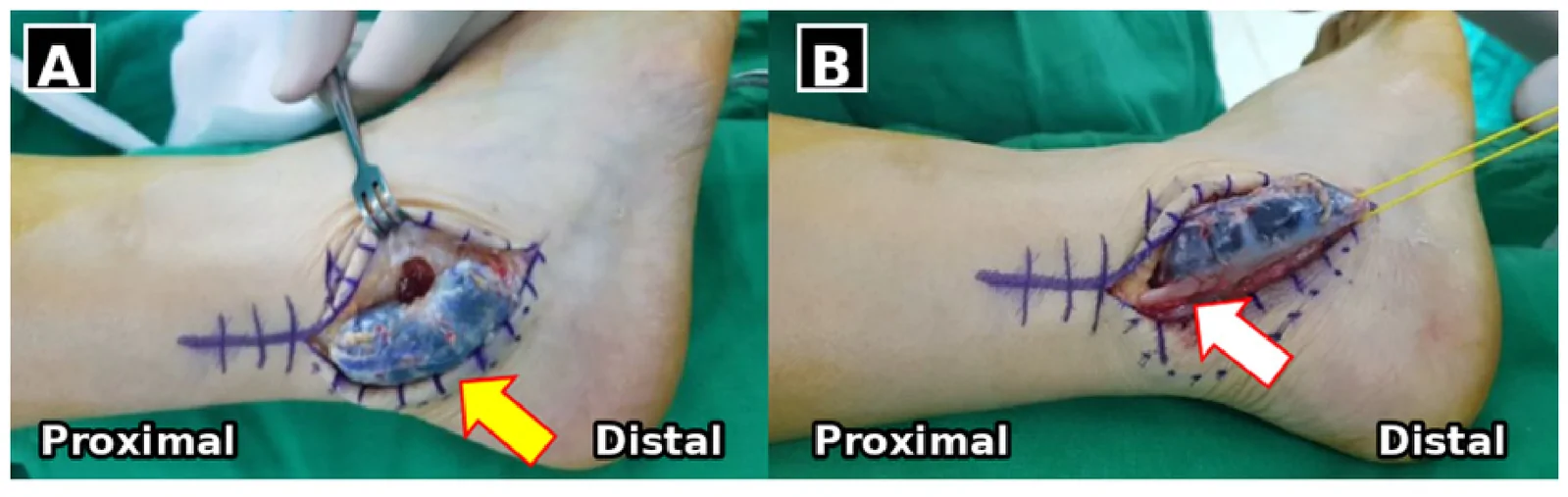
Intraoperative findings of the illustrative surgical case. In both panels, the left side of the image corresponds to the proximal direction and the right side to the distal direction. (**A**) After exposure of the posterolateral ankle, a hemorrhagic cystic lesion (yellow arrow) was identified adjacent to the peroneus quartus (PQ) tendon. (**B**) With further exposure and retraction of the cystic lesion, a distinct PQ tendon separate from the adjacent peroneal tendons was identified (white arrow) and isolated with a vessel loop. The white arrow identifies the PQ tendon; it does not specifically mark the site of tearing. Degenerative tearing with irregular tendon margins was documented in the operative record but is not clearly delineated in the photographs shown. The cystic lesion was subsequently excised, and the tendon was debrided and reconstructed to restore appropriate tension.

Intraoperatively, a distinct accessory tendon, clearly separate from both the peroneus brevis (PB) and peroneus longus (PL) tendons, was identified in the posterolateral ankle and corresponded to the PQ demonstrated on preoperative MRI. The PQ tendon showed a degenerative tear with irregular and disrupted tendon margins. A distended hemorrhagic cystic lesion involving the tendon sheath was observed adjacent to the tear site, and the lesion appeared to be directly associated with the PQ tendon sheath. The PB and PL tendons were intact. Because of the limited extent of surgical exposure, the exact proximal origin and distal insertion of the PQ could not be directly confirmed intraoperatively (Figure 4).

The cystic lesion was excised, and the degenerative and frayed portions of the torn PQ tendon were debrided. Because the remaining PQ tendon was elongated with loss of appropriate tension, it was reconstructed to restore its anatomical course and tension. Tendon reconstruction was performed using a Z-shaped shortening/reconstruction technique, followed by tubularization and core suturing with 2-0 and 3-0 Vicryl sutures. No formal assessment of ankle instability was performed, and no concomitant ligamentous stabilization procedure was undertaken.

Only the excised cystic lesion was submitted for histopathological examination; the debrided tendon tissue was not submitted for pathological evaluation. Histopathological examination of the cystic specimen confirmed a ganglion cyst. Thus, the ganglion cyst was histologically confirmed, whereas the degenerative PQ tendon tear was documented by direct intraoperative observation rather than tendon histopathology.

The patient underwent surgery on 18 December 2018 and was initially followed at approximately 3-month intervals, followed by approximately 6-month intervals thereafter. At the most recent documented follow-up in August 2025, approximately 80 months after surgery, the patient reported satisfaction with the outcome and had no significant recurrent symptoms.

## 4. Discussion

The principal findings were that the PQ was identifiable in 15.4% (95% CI, 11.5–20.2%) of routine ankle MRI examinations in this selected Korean clinical cohort and that two predominant MRI-visible distal attachment patterns were observed. The calcaneal attachment pattern was most common, whereas a smaller proportion joined the PL tendon distally. These findings should be interpreted as MRI-visible morphology in a clinically selected cohort rather than as a population-based prevalence estimate or a complete anatomical classification. The illustrative surgical case demonstrated a symptomatic ganglion in the PQ region; its causal relationship to intrinsic PQ tendon pathology depends on the operative findings described above.

Reported PQ prevalence varies considerably because of differences in study design, population, nomenclature, and detection method. MRI-based studies have reported prevalences ranging from approximately 7% to more than 20% [3,6,10]. More recent large datasets reported 13.8% in 849 ankles [13] and 10.6% in 1160 ankles [14], while the 2026 imaging meta-analysis produced a pooled prevalence of approximately 11% [15]. A recent review continues to emphasize the clinical relevance and imaging recognition of supernumerary muscles of the leg and foot [16], while a recent anatomical investigation demonstrates substantial variability in accessory muscles around the ankle and highlights their relevance to surgical anatomy [18]. Accordingly, the 15.4% prevalence in the present cohort should not be regarded as a novel prevalence estimate in isolation; its value lies in the MRI-based anatomical characterization of this selected cohort and in the availability of 41 PQ-positive examinations for morphologic description.

The numerical proximity between the present MRI prevalence of 15.4% and the 16.3% prevalence reported by Hur et al. in 80 Korean cadaveric lower limbs is of descriptive interest [8], but it should not be interpreted as validation of MRI detection or evidence of population-specific equivalence. The two studies differ in sample selection, demographic composition, examination method, and potentially the unit of analysis. Cadaveric dissection allows direct visualization of small tendinous slips and complex distal insertions, whereas routine ankle MRI is constrained by field of view, slice thickness, tissue contrast, and the difficulty of following very small attachments [11]. Accordingly, patterns not observed on MRI should be distinguished from structures that could not be reliably assessed because of imaging coverage or resolution.

The terminology of accessory peroneal musculature has historically been inconsistent because variants have frequently been named according to their origin or insertion. Terms including peroneocalcaneus externum, peroneocuboideus, and peroneoperoneolongus have been used [1,4,8,10]. Hur et al. proposed a detailed morphological classification based on origin form and distal attachment [8]. The present study was not intended to introduce another formal classification system. Instead, the observed configurations were described according to their MRI-visible distal attachments and interpreted in relation to established anatomical terminology.

The calcaneal insertion pattern accounted for 85.4% of PQ-positive examinations (95% CI, 71.6–93.1%) and corresponded most closely to the historically described peroneocalcaneus externum. The PL attachment pattern accounted for 14.6% (95% CI, 6.9–28.4%) and resembled the peroneoperoneolongus configuration. The wide confidence interval for the PL attachment pattern reflects the small number of observations and warrants cautious interpretation. The relative simplicity of these MRI-visible patterns should not be interpreted as evidence that only two PQ variants exist; rather, it describes the configurations most readily recognizable with the routine ankle MRI protocols used in this study.

Most PQ muscles are incidental and asymptomatic, and identification on MRI does not itself establish pathology [11,15]. Nevertheless, the anatomical concept of retromalleolar crowding provides a plausible mechanism for the association between an additional musculotendinous structure and peroneal tendon disorders. Earlier cadaveric and clinical reports proposed relationships with PB longitudinal tears, tendon subluxation, tenosynovitis, and lateral ankle pain [2,5,7]. Evidence regarding this association was historically inconsistent. Yammine’s 2015 meta-analysis did not demonstrate convincing evidence that accessory peroneal musculature was systematically associated with peroneal tendon pathology [10]. In contrast, Yuksel et al. reported increased odds of PB tendinitis (OR 3.06), PB tear (OR 3.64), and PL tendinitis (OR 2.43) in PQ-positive ankles [14]. The subsequent 2026 imaging meta-analysis reported a pooled odds ratio of 3.40 for peroneal tendon tear in ankles with a PQ [15]. These findings support the concept that PQ may act as a predisposing anatomical factor in selected patients, although causality remains unproven.

Previous clinical reports have focused on the effects of the PQ on adjacent peroneal structures [5,7,16], whereas other reports have highlighted its potential use as local reconstructive tissue [7,17]. In the illustrative patient, MRI demonstrated a cystic lesion adjacent to the PQ tendon and suggested tendon degeneration; intraoperatively, a degenerative PQ tendon tear with irregular margins was directly observed, and a hemorrhagic cystic lesion involving the adjacent tendon sheath was documented. Histopathology confirmed the excised lesion as a ganglion cyst, whereas tendon tissue was not submitted for histological examination. Thus, the case documents a symptomatic ganglion anatomically associated with an operatively observed PQ tendon tear, but a causal relationship between the PQ variant, the ganglion, and the patient’s symptoms cannot be established from a single case.

Recognition of the PQ is also relevant during surgical treatment of peroneal tendon disorders. Symptomatic accessory musculature contributing to crowding may require decompression or excision in selected patients [5,7,11]. Conversely, the PQ tendon may provide locally available autologous tissue; Bilgili et al. described its use for superior peroneal retinaculum reconstruction [7], and Cossogue et al. reported augmentation of PB longitudinal tear repair using the PQ tendon [17]. Thus, preoperative recognition may be useful both for identifying a potential contributor to pathology and for anticipating an anatomical structure that may be encountered during reconstruction.

### Limitations

Several limitations should be acknowledged. First, this was a retrospective imaging-based study of a selected clinical referral cohort rather than a population-based sample. All patients underwent MRI for suspected Achilles tendon injury, and the marked male predominance (90.6%) further limits generalizability, particularly to female patients. Second, although the final 267 observations represented unique patients and unique ankles without bilateral or repeated examinations, routine clinical MRI cannot reproduce the spatial resolution or direct structural confirmation provided by anatomical dissection. The proximal origin of a PQ may fall outside the ankle MRI field of view, and thin distal attachments may be difficult to follow; therefore, directly visualized anatomy must be distinguished from unconfirmed or indeterminate structures. Third, systematic clinical information regarding lateral ankle symptoms and concomitant peroneal tendon abnormalities was not available for every patient, and the study was not designed to compare peroneal pathology between PQ-positive and PQ-negative groups. Accordingly, no causal association between PQ presence or morphology and tendon disease can be inferred. Fourth, no morphometric measurements of the PQ or retromalleolar tunnel were performed. Fifth, surgical and histopathologic correlation was available in only one illustrative patient, and histologic confirmation of a ganglion cyst does not by itself establish intrinsic PQ tendon pathology. Sixth, because the same readers had previously assessed these examinations, familiarity with the cases may have influenced agreement during re-review. Finally, the relatively small number of PQ-positive examinations, particularly the six PL attachment cases, results in statistical uncertainty reflected by the corresponding confidence intervals. Future prospective studies using standardized imaging coverage, predefined clinical assessments, quantitative retromalleolar measurements, dynamic imaging, and operative correlation may better define the clinical significance of individual PQ configurations.

## 5. Conclusions

The peroneus quartus was identified in 15.4% of ankle MRI examinations in this selected Korean clinical cohort, with retrotrochlear calcaneal insertion and distal confluence with the peroneus longus tendon representing the two predominant MRI-visible patterns. Recognition of these variants may improve MRI interpretation and the surgical assessment of posterolateral ankle pathology. The illustrative surgical case suggests that the PQ tendon and its sheath may themselves become symptomatic, although no causal inference can be drawn from a single case.

## Figures and Tables

**Table 1:** Study cohort and MRI findings of the peroneus quartus. PQ, peroneus quartus.

Variable	Finding
Initially selected ankle MRIs	300
Excluded examinations	33
Included ankle MRIs	267
Mean age	43.6 ± 10.6 (20–75)
Male/female	242 (90.6%)/25 (9.4%)
Right/left	162 (60.7%)/105 (39.3%)
PQ present	41/267 (15.4%; 95% CI, 11.5–20.2%)
Calcaneal insertion pattern	35/41 (85.4%; 95% CI, 71.6–93.1%)
Peroneus longus insertion pattern	6/41 (14.6%; 95% CI, 6.9–28.4%)

**Table 2:** Comparison of MRI-visible PQ morphology in the present cohort with representative anatomical descriptions in the literature. PB, peroneus brevis; PL, peroneus longus; PQ, peroneus quartus.

Anatomical Configuration	Previous Terminology/Description	Representative Literature	Present MRI Cohort	Interpretation
Calcaneal attachment	Peroneocalcaneus externum; retrotrochlear calcaneal insertion	Cheung et al. [3]; Yammine [10]	35/41 (85.4%)	Directly visualized distal attachment
PL tendon attachment	Peroneoperoneolongus/accessory peroneal tendon joining PL	Clarkson et al. [4]; Yammine [10]	6/41 (14.6%)	Direct tendon continuity required
Other reported distal attachments	Cuboid, fifth metatarsal, PB tendon, inferior retinaculum, peroneal trochlea	Clarkson et al. [4]; Hur et al. [8]; Borthakur et al. [9]	Not observed	Absence in this MRI cohort does not exclude anatomical occurrence
Proximal origin: PB	PB is a commonly reported origin	Hur et al. [8]; Yammine [10]	19/41: calcaneal 18; PL 1	Origin directly visualized
Proximal origin: distal fibula	Fibular origin has also been reported	Hur et al. [8]; Yammine [10]	13/41: calcaneal 12; PL 1	Origin directly visualized
Proximal origin: not directly visualized	—	—	9/41: calcaneal 5; PL 4	Proximal extent lay outside the MRI field of view; origin was not inferred

## Data Availability

The datasets used and/or analysed during the current study are available from the corresponding author on reasonable request.

## Supplementary Materials

The following supporting information can be downloaded at: https://www.mdpi.com/article/10.3390/jcm15197732/s1, Table S1: Interobserver agreement for PQ detection and distal attachment.
