# Supplementary material for: MRI-Based Morphologic Patterns of the Peroneus Quartus in a Korean Clinical Cohort: An Anatomical Study with an Illustrative Surgical Case
Source: J Clin Med. 2026 Oct 8;15(19):7732. doi: 10.3390/jcm15197732 (PMC13665722; doi:10.3390/jcm15197732)
Supplement: Supplementary file 1 [file jcm-15-07732-s001.zip › jcm-4565143-supplementary.pdf]

Supplementary Table S1. Interobserver agreement for PQ detection and distal attachment

PQ detection	Reader 2 positive	Reader 2 negative	Distal attachment	Reader 2 calcaneal	Reader 2 PL
**Reader 1 positive**	41	0	**Reader 1 calcaneal**	35	0
**Reader 1 negative**	0	226	**Reader 1 PL**	0	6

Note: PQ detection: observed agreement, 100%; Cohen's κ = 1.00. Distal attachment classification: observed agreement, 100%; Cohen's κ = 1.00. Asymptotic 95% confidence intervals are not reported because complete agreement yielded degenerate intervals of 1.00–1.00. Seven distal attachment assessments (three PL and four calcaneal) were flagged as lower-confidence by one reader; joint review did not change their classifications.
